# Advances in the Monitoring, Diagnosis and Optimisation of Water Systems

**DOI:** 10.3390/s23063256

**Published:** 2023-03-20

**Authors:** Miquel Àngel Cugueró-Escofet, Vicenç Puig

**Affiliations:** 1 Advanced Control Systems (SAC) Research Group, Polytechnic University of Catalonia (UPC-Barcelo-naTech), Terrassa Campus, Gaia Research Bldg, Rambla Sant Nebridi, 22, 08222 Terrassa, Spain; 2 Institut de Robòtica i Informàtica Industrial (CSIC-UPC), 46 Llorens i Artigas Street, 08028 Barcelona, Spain

In the context of global climate change, with the increasing frequency and severity of extreme events—such as draughts and floods—which will likely make water demand more uncertain and jeopardise its availability, those in charge of water system management face new operational challenges because of increasing resource scarcity, intensive energy requirements, growing populations (especially in urban areas), costly and ageing infrastructures, increasingly stringent regulations, and rising attention towards the environmental impact of water use. The shift from a linear to a circular economy and the need for a transition to a low-carbon production system represents an opportunity to address these emerging challenges related to water, energy, and the efficient use of resources. These challenges impel network managers to improve their methods and techniques for the monitoring, diagnosis, prognosis, supervision, and optimisation of the performance of water-related systems to adhere to the current sustainability agenda.

In this context, the increasing number of advanced installed sensors—and the corresponding increase in available data—allow for the implementation of Industry 4.0 (I4.0) techniques, which are strongly focused on interconnectivity, automation, artificial intelligence (AI), and real-time data acquisition, and will facilitate the development of intelligent tools to tackle such challenges. Within this framework, the successful implementation of I4.0 techniques in water-cycle-management facilities may prompt a breakthrough in improving the processes involved, drastically increasing their performance.

In this Special Issue, a selection of these techniques applied to the integral water cycle—i.e., water distribution and water sanitation—is introduced to address different current water-management challenges. These challenges may be classified as water-quantity challenges and water-quality challenges. On the water-distribution side, these challenges may include fault detection—namely, leak localisation—in water-distribution networks (WDNs), e.g., in [1], where a process prior to the actual leak localisation—i.e., sensor placement—is carried out using information-theory simulation-based methodology; or in [2], where a new data-driven method for leak location considering pressure measurements and network topological information is presented; or in [3], where simultaneous leak detection and isolation is applied to real data. All these methodologies contribute to reducing water loss due to leaks, which may account for up to 65% of the total water depending on the network [3] and, hence, impact water-quantity-management challenges. WDNs are also the focus in [4], where a challenge from the water-quality side—particularly, the water-disinfection process in water distribution—is addressed, providing a water-quality model by an online chlorine-decay-model calibration method, which has a strong impact on human health, since its correct concentration is paramount to ensure safe water disinfection.

Work presented in [5,6,7,8] discusses the water-sanitation side. In this field, there is a growing interest in the adaptation and use of technologies related to the circular economy which promote environmental sustainability, where resource recovery is a key issue for industrial and environmental processes and involves a wide spectrum of study possibilities. In water sanitation, wastewater treatment plants (WWTPs) offer a wide range of possibilities for resource recovery, mainly related to sludge-treatment processes such as biogas generation via the substrate codigestion process, which can be an alternative source for thermal and electrical energy production. This potential for biogas generation could become a source of renewable natural gas, which has specific composition requirements that demand high-tech sensors to assure its quality no matter its origin. Due to their potential for resource recovery and the further implications in the water–food–energy nexus, WWTPs have been a research focus in different areas of expertise: from modelling and engineering design to process dynamics, simulation, and integration. This line of work is introduced in [6], where resource recovery—namely biogas in the latter reference—is optimised by a centralised codigestion method considering real data from a WWTP network. Different nature-inspired optimisation algorithms are compared in the performance of this task, providing potential dramatic improvement when compared with actual nonoptimised operation. The improved operation of WWTP is also sought in [5,7] by means of improving the controllers involved in the operation of certain key processes of the WWTP, e.g., the aeration process of biological reactors. Classic proportional-integral (PI) controllers have been traditionally considered as the control strategy for such processes; however, improved performance may be achieved with more complex structures and techniques, e.g., model predictive-control (MPC) schemes or artificial neural network (ANN) approaches. In [5], an economic MPC (EMPC) considering a linear parameter-varying (LPV) model is proposed to control dissolved oxygen concentration in the WWTP biological reactors. Since the MPC technique requires a model of the process involved for its control, in the latter reference, a reduced model of the complex nonlinear plant is represented in a quasilinear parameter-varying (qLPV) form to reduce the computational burden—enabling the real-time operation—and applied in a real facility. This model, however, may be not available or may be difficult to obtain since the processes involved in the WWTP include nonlinear relations. ANN schemes may provide an alternative to this issue since they are well suited to deal with such processes. In this line of work, [7] considers transfer-learning (TL) methods to train ANN nets supporting control operations in WWTPs, and compares this approach with traditional control schemes, providing improved control performance while reducing control-design complexity and time invested in the ANN training process, which can be considerably time-demanding. Last but not least, in this Special Issue collection, a soft-sensing approach to predict key performance indicators (KPIs) in water-quality monitoring and control of WWTPs—such as effluent biochemical oxygen demand (BOD) or ammonia nitrogen (NH3-N)—is presented in [8]. Water-quality KPIs in WWTPs are traditionally subject to nonautomated lab-based offline monitoring approaches. Instead, in the latter reference, a method to perform accurate predictions of these KPIs, aiming for online operation, is introduced.

Further work in this area is included in the Special Issue, e.g., in [9], where remote sensing (RS) image-based time series are considered to obtain mass balances and estimate the unfiltered volumes in topographic depressions which are seasonally filled with water in a real area; or in [10], where a soil-moisture monitoring technique in precision agriculture—which is becoming key to providing food sustainably in the context of world’s increasing population and natural resource scarcity—is provided using a low-cost wireless sensor network in order to help farmers optimise the irrigation process, and is tested in a real plot of land. Finally, a comprehensive review of AI and computer-vision methods for intelligent water monitoring—namely, water-body extraction and water-quality monitoring—using RS techniques is presented in [11], discussing the main challenges of using AI and RS for water-information extraction, as well as pointing out research priorities in this area. Hence, all the contributions in this Special Issue have an impact on the advances in the monitoring, diagnosis, and optimisation of water systems and, overall, cover a wide and complete sector of knowledge within this area.

## References

[1] Santos-Ruiz I., López-Estrada F.R., Puig V., Valencia-Palomo G., Hernández H.R. (2022). Pressure Sensor Placement for Leak Localization in Water Distribution Networks Using Information Theory. Sensors.

[2] Alves D., Blesa J., Duviella E., Rajaoarisoa L. (2021). Robust data-driven leak localization in water distribution networks using pressure measurements and topological information. Sensors.

[3] Navarro-Díaz A., Delgado-Aguiñaga J.A., Begovich O., Besançon G. (2021). Two simultaneous leak diagnosis in pipelines based on input–output numerical differentiation. Sensors.

[4] Pérez R., Martínez-Torrents A., Martínez M., Grau S., Vinardell L., Tomàs R., Martínez-Lladó X., Jubany I. (2022). Chlorine Concentration Modelling and Supervision in Water Distribution Systems. Sensors.

[5] Nejjari F., Khoury B., Puig V., Quevedo J., Pascual J., de Campos S. (2022). Economic Linear Parameter Varying Model Predictive Control of the Aeration System of a Wastewater Treatment Plant †. Sensors.

[6] Palma-Heredia D., Verdaguer M., Puig-Cayuela V., Poch M., Cugueró-Escofet M.À. (2022). Comparison of optimisation algorithms for centralised anaerobic co-digestion in a real river basin case study in Catalonia. Sensors.

[7] Pisa I., Morell A., Vilanova R., Vicario J.L. (2021). Transfer learning in wastewater treatment plant control design: From conventional to long short-term memory-based controllers. Sensors.

[8] Zhou M., Zhang Y., Wang J., Shi Y., Puig V. (2022). Water Quality Indicator Interval Prediction in Wastewater Treatment Process Based on the Improved BES-LSSVM Algorithm. Sensors.

[9] Fil P.P., Yurova A.Y., Dobrokhotov A., Kozlov D. (2021). Estimation of infiltration volumes and rates in seasonally water-filled topographic depressions based on remote-sensing time series. Sensors.

[10] Lloret J., Sendra S., Garcia L., Jimenez J.M. (2021). A wireless sensor network deployment for soil moisture monitoring in precision agriculture. Sensors.

[11] Yang L., Driscol J., Sarigai S., Wu Q., Lippitt C.D., Morgan M. (2022). Towards Synoptic Water Monitoring Systems: A Review of AI Methods for Automating Water Body Detection and Water Quality Monitoring Using Remote Sensing. Sensors.

